# Treatment of Fracture of the Clavicle by Suturing the Fractured Ends

**Published:** 1882-04

**Authors:** 


### TREATMENT OF FRACTURE OF THE CLAVICLE BY SUTURING THE FRACTURED ENDS.

Langenbuch (*Deutsche Med. Wochenschr.*, No. 5, 1882) gives an account of a case of fracture of the clavicle between the middle and external third of the bone, which he treated by suturing the fractured ends. An incision was made down the bone through the periosteum, which had remained uninjured. A complete transverse fracture of the bone was found, with displacement downward and backward of the sternal end, which was exceedingly mobile. The ends of the bone were raised by means of hooks and isolated from the underlying tissues by metallic strips. After disinfecting the wound, the fractured ends were perforated with a drill and a silver wire passed through and twisted, bringing the bones into exact coaptation. The ends of the wires were turned down into one of the holes in the bone, and the periosteum united with catgut. The wound of the integument was closed with silk sutures. No drainage. Antiseptic gauze. A few turns of a roller on the principle of Desault, to fix the arm, completed the dressing. An ideal recovery followed. [Langenbuch does not state the time in which union was accomplished.—Tr.]

